# Micromachined Vibrating Ring Gyroscope Architecture with High-Linearity, Low Quadrature Error and Improved Mode Ordering

**DOI:** 10.3390/s20154327

**Published:** 2020-08-03

**Authors:** Zezhang Li, Shiqiao Gao, Lei Jin, Haipeng Liu, Shaohua Niu

**Affiliations:** 1 State Key Laboratory of Explosion Science and Technology, Beijing Institute of Technology, Beijing 100081, China; zezhang_li@163.com (Z.L.); lhp@bit.edu.cn (H.L.); shh@bit.edu.cn (S.N.); 2 School of Mechatronical Engineering, Beijing Institute of Technology, Beijing 100081, China; jinlei@bit.edu.cn

**Keywords:** vibrating ring gyroscope, high-linearity, quadrature error, improved mode ordering, hinge-frame mechanism

## Abstract

A new micromachined vibrating ring gyroscope (VRG) architecture with low quadrature error and high-linearity is proposed, which successfully optimizes the working modes to first order resonance mode of the structure. The improved mode ordering can significantly reduce the vibration sensitivity of the device by adopting the hinge-frame mechanism. The frequency difference ratio is introduced to represent the optimization effect of modal characteristic. Furthermore, the influence of the structural parameters of hinge-frame mechanism on frequency difference ratio is clarified through analysis of related factors, which contributes to a more effective design of hinge-frame structure. The designed VRG architecture accomplishes the goal of high-linearity by using combination hinge and variable-area capacitance strategy, in contrast to the conventional approach via variable-separation drive/sense strategy. Finally, finite element method (FEM) simulations are carried out to investigate the stiffness, modal analysis, linearity, and decoupling characteristics of the design. The simulation results are sufficiently in agreement with theoretical calculations. Meanwhile, the hinge-frame mechanism can be widely applied in other existing ring gyroscopes, and the new design provides a path towards ultra-high performance for VRG.

## 1. Introduction

Due to the advantages on miniaturization, lightweight, easy integration, mass-production, and low-power consumption, the micro-electro-mechanical system (MEMS) vibrating gyroscope has become a significantly attractive device in a wide range of applications including automotive industry, biotechnology, medicine, space as well as consumer electronics [1,2,3]. Compared with tuning fork gyroscope (TFG), vibrating ring gyroscope (VRG) has the following advantages: (1) the sensitivity of the sensor is amplified by the quality factor due to the driving and sensing modes having identical frequency when the structures are fabricated from isotropic materials [4,5]. (2) It is less sensitive to temperature variations because thermal environment affects the identical vibrational modes of resonant rings equally [6,7]. (3) It is robust against random vibration since external vibrations do not couple to the identical vibration modes of rings [4,7,8,9].

A typical VRG consists of a ring structure, support beams and electrodes surrounding the ring structure. The electrodes are used for drive, sense, or control of the gyro. The operation of the ring gyroscope relies on two elliptically shaped vibration modes (flexural modes) [4,5,9,10,11,12,13,14,15]. The study of VRG has been widely reported in the literature. Michael William Putty et al. firstly developed and fabricated the VRG with an open-loop sensitivity of 200 μV/°/s in a dynamic range of ±250 °/s under a low vacuum level [4,5]. Based on the design concept mentioned above, several variations of the architecture were also reported in the literature [10,11,12,13,14,15]. Meanwhile, much effort was already made to improve the gyroscope performance, including high shock reliability [7,9,16], parametric amplification [17,18,19], electrostatic tuning [20,21,22] and so on.

It is obvious that the limited dynamic range of VRGs (around ±250 °/s) mentioned above is insufficient to meet the demand for tactical applications. The original sources of the limited full-scale range are the quadrature error and the parallel-plate sensing mechanism assumed to be linear consisting of fixed electrodes and the outer circumference of the ring. The device vibration is mostly linear at normally used levels, however, the nonlinear jump that occurs at higher vibration level will restrict the application of ring gyroscope in higher requirements [4]. Some researchers focus on modelling of nonlinearities and control of nonlinear vibration [23,24,25,26,27,28]. On the other hand, much work has been done in decoupling the drive and sense modes, in order to minimize the quadrature error. The quadrature error can be largely eliminated at low vibration levels [20,21,22], while the drive/sense strategy remains the same and the improvement of the decoupling is extremely limited.

The vibration-induced error caused by poor mode ordering not only exists in TFG but also in VRG [29,30,31]. The resonant frequencies of translation modes in previous ring gyroscope designs are much smaller than those of the flexural modes (i.e., working modes). Detailed theoretical derivation by Sang Won Yoon et al. demonstrated that ring gyroscopes with non-proportional damping were not immune from external vibration. Therefore, the translation modes are more susceptible to external/environmental vibration whose frequency spectrum also lies well below resonant frequencies of flexural modes [8,32].

The hinge mechanisms with the advantage of rotation characteristic have attracted much attention and applied in MEMS devices, such as MEMS TFG, MEMS accelerometer and ring coupled gyroscope (RCG) [33,34,35]. The in-plane, n = 3 mode RCG designs of hinge mechanism have been adopted to increase the capacitive transduction areas by means of the auxiliary arrayed transducers. However, little attention has been paid to the principle of mode ordering improvement of VRG with hinge mechanisms. Besides, hinge mechanism on the optimization of decoupling and linearity of VRG is rarely reported.

In this paper, a novel designed vibrating ring gyroscope with a hinge-frame mechanism (HFVRG) is proposed. The structural characteristics and advantages of a HFVRG are introduced in Section 2. Section 3 establishes dynamic equations and discusses the modal characteristics as well as theoretical calculation. Finite element method (FEM) simulation of the stiffness of different springs and comparisons between simulation and theoretical results are discussed, and the linearity of the design is analyzed in Section 4. In Section 5, the discussions are given. Section 6 concludes the paper with a summary.

## 2. Architecture Design

**Figure 1:**
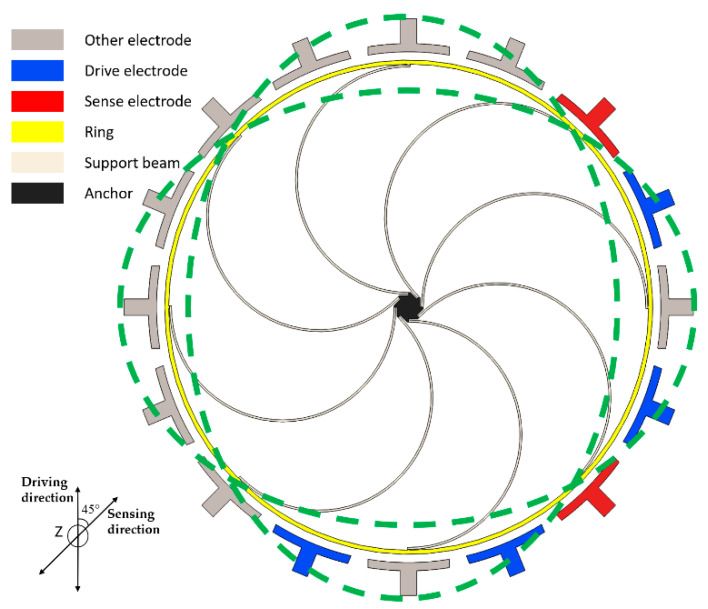
Schematic view of a typical vibrating ring gyroscope.

The typical VRG, shown in Figure 1, consists of a ring structure, eight semicircular elastic support beams and electrodes. These electrodes can be sense, drive or control electrodes. Under the normal working condition of the VRG, the ring structure is forced to vibrate at the drive mode resonant frequency of the device along the green imaginary line. At higher vibration level, nonlinear jump phenomenon in sense capacitance of the sense electrode will lead to a stronger nonlinear response of the whole system.

**Figure 2:**
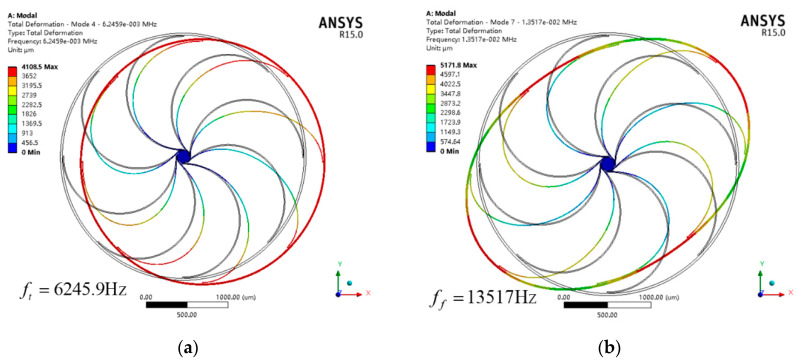
Modal analysis of the translation mode (**a**) and flexural mode (**b**) of a typical vibrating ring gyroscope.

As mentioned above, typical VRG designs that possess translation modes have natural frequencies that are much smaller than those of flexural modes. As an intuitive quantitative consideration, Workbench is used to simulate the flexural mode and translation mode of the typical VRG shown in Figure 1 with radius, height and width of 1500 μm, 80 μm and 25 μm, respectively. The simulation results in Figure 2 illustrate that the frequencies of translation modes lie well below those of the flexural modes. Undoubtedly, this ring gyroscope is more impressionable to environmental vibration.

**Figure 3:**
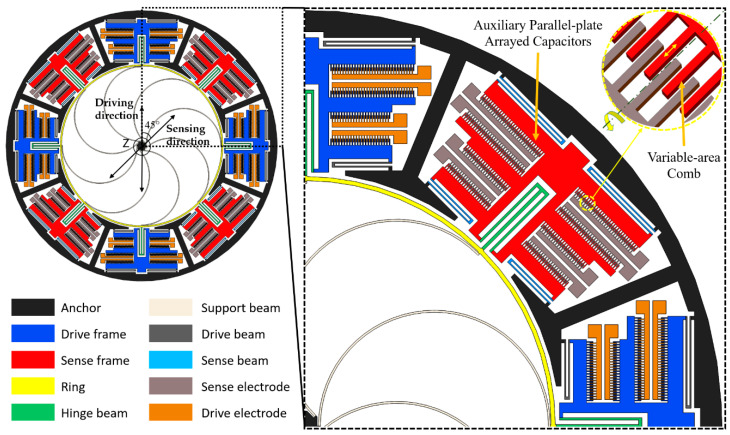
Schematic of vibrating ring gyroscope with a hinge-frame mechanism.

In order to eliminate the effects of nonlinear response and poor mode ordering, a novel vibrating ring gyroscope with a hinge-frame mechanism as drive/sense frame is designed, see Figure 3. The HFVRG is mainly composed of a ring structure, 8 semicircular elastic support beams, 8 hinge beams, 32 drive/sense beams, 8 drive/sense frames, and 8 groups of drive/sense electrodes. It is noteworthy that the resonant ring and support beams of HFVRG are identical to those of the typical VRG in Figure 1. The advantages of this design include:

(1). The hinge beams can realize the rotation function and enable effect decouple between the drive mode and sense mode. The capacitance nonlinearity generated by capacitance change between the ring and the pickoff electrode in a typical VRG can be eliminated through variable-area comb mechanism.

(2). The hinge beams are located at the maximum radial displacement of the resonant ring to realize electromechanical coupling efficiently between the mechanical ring vibration and the electrical signal of drive/sense electrodes. Furthermore, the drive/sense electrodes with auxiliary parallel-plate arrayed capacitors are adopted to increase the capacitive transduction areas remarkably.

(3). The geometric symmetry structure between the ring and the anchor contributes to releasing the structural stress in the fabrication process.

## 3. Theoretical Analysis

The HFVRG operates as follows. The primary flexural (drive) mode is driven into resonance by driving voltages imposed on the drive electrodes of variable-area along the driving direction. When the device is subjected to rotation, a proportion of the vibration energy is converted from the primary flexural (drive) mode to the secondary flexural (sense) mode. Then vibration occurs in the sense direction due to the Coriolis effect. The amplitude of movable parallel plate displacement on the sense frame is proportional to the angular rate. Based on the principle, the angular rate of rotation is measured by detecting the displacement. As is depicted in Figure 3.

The stiffness of the support beams, hinge beams and drive/sense beams in the design of this paper are much less than those of drive/sense frames. On the other hand, the mass of the frames is larger than that of the beams. Therefore, the drive/sense frames are simplified to inelastic mass. Considering that the stiffness and mass of the vibrating ring structure are distributed along the ring, each beam is represented as a discrete spring attached to the drive/sense frame or ring mass and the ring is represented as a continuous (curved) beam.

### 3.1. Normal Modes of HFVRG

As described above, the MEMS HFVRG has two working modes: the primary flexural (drive) mode and the secondary flexural (sense) mode. Additionally, a more common translational vibration induced by environmental vibrations may also excite other in-the-plane modes. Particularly, the two modes of ring translation cause a rigid translation of the ring on its support beams. Therefore, the overall motion of the ring can be decomposed into the motions that are excited by the gyroscope’s operation (flexural modes) and the environment vibration (in-the-plane translation modes). In the present model, it was assumed that the ring performs in-plane motion only, and the out of plane motion was neglected [8,32].

Vibrating ring structure can be analyzed using normal modes. This analysis strategy is based on the fact that any plane vibration-induced displacement of the ring u can be demonstrated as the linear combination of its flexural modes ($\varphi_{1}$ and $\varphi_{2}$) and translation modes ($\varphi_{3}$ and $\varphi_{4}$). The total displacement of the ring structure is given by:

(1) $$u=u\left(t,\theta \right)=\sum_{i=1}^{4}\varphi_{i}\left(\theta \right)q_{i}\left(t\right)$$

where θ is the independent spatial coordinate(s), and $q_{i}(t)$ are the generalized (modal) coordinates of the dynamic model of HFVRG.

The horizontal and vertical displacement of ring($u_{x}$ and $u_{y}$) are governed by:

(2) $$\begin{array}{l} u_{x}=\varphi_{x1}q_{1}+\varphi_{x2}q_{2}+\varphi_{x3}q_{3}+\varphi_{x4}q_{4}\\ u_{y}=\varphi_{y1}q_{1}+\varphi_{y2}q_{2}+\varphi_{y3}q_{3}+\varphi_{y4}q_{4} \end{array}$$

where Equation (2) is the Cartesian coordinate form of Equation (1). $\varphi_{x1}$, $\varphi_{x2}$, $\varphi_{x3}$, $\varphi_{x4}$, $\varphi_{y1}$, $\varphi_{y2}$, $\varphi_{y3}$, and $\varphi_{y4}$ are the horizontal and vertical components of four plane modes in Cartesian coordinate system.

The mode shapes of a ring structure can be derived from simple calculations taking advantage of the symmetry of the ring structure [32]. The two elliptical-shaped flexural modes and two in-the-plane translation modes with Cartesian components are given by:

(3) $$\mathrm{Drive}-\mathrm{axis}\;\mathrm{flexural}\;\mathrm{mode}:\varphi_{x1}=\left[\frac{1}{4}\cos\left(3\theta \right)+\frac{3}{4}\cos\left(\theta \right)\right],\;\varphi_{y1}=\left[\frac{1}{4}\sin\left(3\theta \right)-\frac{3}{4}\sin\left(\theta \right)\right]$$

(4) $$\mathrm{Sense}-\mathrm{axis}\;\mathrm{flexural}\;\mathrm{mode}:\varphi_{x2}=\left[\frac{1}{4}\sin\left(3\theta \right)+\frac{3}{4}\sin\left(\theta \right)\right],\;\varphi_{y2}=\left[-\frac{1}{4}\cos\left(3\theta \right)+\frac{3}{4}\cos\left(\theta \right)\right]$$

(5) $$X-\mathrm{axis}\;\mathrm{translation}\;\mathrm{mode}:\varphi_{x3}=1,\;\varphi_{y3}=0$$

(6) $$Y-\mathrm{axis}\;\mathrm{translation}\;\mathrm{mode}:\varphi_{x4}=0,\;\varphi_{y4}=1$$

### 3.2. System Equations of HFVRG

The system equations of motion of HFVRG are derived in the literature by formulating the mechanical potential energy, kinetic energy, electrical potential energy, and energy dissipation by viscous damping, and employing Lagrange’s method [8,32]. The four degree-of-freedom dynamical equation is as follows.

(7) $$\begin{array}{ccc} mass & damping & Coriolis\;couping\\ \left[\begin{array}{cccc} M_{1} & & & \\ & M_{2} & & \\ & & M_{3} & \\ & & & M_{4} \end{array}\right] & \left\{\begin{array}{c} {\ddot{q}}_{1}\\ {\ddot{q}}_{2}\\ {\ddot{q}}_{3}\\ {\ddot{q}}_{4} \end{array}\right\}+\left[\begin{array}{cccc} c_{11} & & & \\ & c_{22} & & \\ & & c_{33} & \\ & & & c_{44} \end{array}\right]\left\{\begin{array}{c} {\dot{q}}_{1}\\ {\dot{q}}_{2}\\ {\dot{q}}_{3}\\ {\dot{q}}_{4} \end{array}\right\}+ & \left[\begin{array}{cccc} 0 & -2\gamma_{1}\Omega_{z} & & \\ 2\gamma_{1}\Omega_{z} & 0 & & \\ & & 0 & -2\gamma_{2}\Omega_{z}\\ & & 2\gamma_{2}\Omega_{z} & 0 \end{array}\right]\left\{\begin{array}{c} {\dot{q}}_{1}\\ {\dot{q}}_{2}\\ {\dot{q}}_{3}\\ {\dot{q}}_{4} \end{array}\right\}+ \end{array}\;\begin{array}{cccc} angular\;acceleration & centripetal\;stiffness & stiffness & drive\;force\\ \left[\begin{array}{cccc} 0 & -\gamma_{1}{\dot{\Omega }}_{z} & & \\ \gamma_{1}{\dot{\Omega }}_{z} & 0 & & \\ & & 0 & -\gamma_{2}{\dot{\Omega }}_{z}\\ & & \gamma_{2}{\dot{\Omega }}_{z} & 0 \end{array}\right] & \left\{\begin{array}{c} q_{1}\\ q_{2}\\ q_{3}\\ q_{4} \end{array}\right\}-\Omega_{z}^{2}\left[\begin{array}{cccc} M_{1} & & & \\ & M_{2} & & \\ & & \alpha_{1} & \\ & & & \beta_{2} \end{array}\right]\left\{\begin{array}{c} q_{1}\\ q_{2}\\ q_{3}\\ q_{4} \end{array}\right\}+ & \left[\begin{array}{cccc} k_{1} & & & \\ & k_{2} & & \\ & & k_{3} & \\ & & & k_{4} \end{array}\right] & \left\{\begin{array}{c} q_{1}\\ q_{2}\\ q_{3}\\ q_{4} \end{array}\right\}=\left\{\begin{array}{c} f_{1}\\ f_{2}\\ f_{3}\\ f_{4} \end{array}\right\} \end{array}$$

where

(8) $$M_{1}=\int_{V}\rho \left[{\left(\varphi_{x1}\right)}^{2}+{\left(\varphi_{y1}\right)}^{2}\right]dV+4m_{f},\;M_{2}=\int_{V}\rho \left[{\left(\varphi_{x2}\right)}^{2}+{\left(\varphi_{y2}\right)}^{2}\right]dV+4m_{f}\;M_{3}=\int_{V}\rho \left[{\left(\varphi_{x3}\right)}^{2}+{\left(\varphi_{y3}\right)}^{2}\right]dV+2m_{f},\;M_{4}=\int_{V}\rho \left[{\left(\varphi_{x4}\right)}^{2}+{\left(\varphi_{y4}\right)}^{2}\right]dV+2m_{f}$$

(9) $$\gamma_{1}=\int_{V}\rho \left[\varphi_{x3}\varphi_{y3}\right]dV,\;\gamma_{2}=\int_{V}\rho \left[\varphi_{x3}\varphi_{y4}-\varphi_{x4}\varphi_{y3}\right]dV,\;\alpha_{1}=\int_{V}\rho {\left(\varphi_{x3}\right)}^{2}dV,\;\beta_{2}=\int_{V}\rho {\left(\varphi_{y4}\right)}^{2}dV$$

(10) $$K_{F}=K_{ri}+4K_{hs}+8K_{dsy1}+8K_{dsy2},\;K_{T}=2K_{hs}+2K_{vs}+4K_{45}+2K_{hx}+4K_{dsy1}+4K_{dsy2}$$

(11) $$K_{ri}=10\pi \frac{EI}{r_{r}^{3}}\left(i=1,2\right),\;K_{ns}=\frac{2\pi EI_{s}}{\left(\pi^{2}-8\right)r_{s}{}^{3}},K_{ts}=\frac{2EI_{s}}{\pi r_{s}{}^{3}},K_{45}=\frac{2\left(\pi^{2}-4\right)EI_{s}}{\pi \left(\pi^{2}-8\right)r_{s}{}^{3}}$$

**Figure 4:**
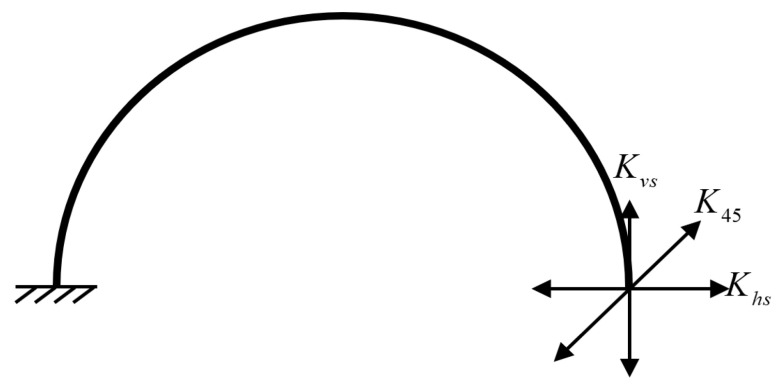
Stiffnesses of a semicircular elastic support beam in three directions.

Here, $M_{i}(i=1,2)$ describes the modal mass associated with the flexural modes of HFVRG. $m_{f}$ is the mass of the drive/sense frame. ρ and V represent the density and volume of the ring structure, respectively. $M_{i}(i=3,4)$ describes the modal mass associated with the translation modes of the architecture. The quantities $\gamma_{1}$ and $\gamma_{2}$ denote respectively a modal coupling induced by the Coriolis force and angular acceleration, and the quantities $\alpha_{1}$ and $\beta_{2}$ indicate additional stiffness induced by the centripetal acceleration. $c_{ii}\left(i=1,2,3,4\right)$ are the modal damping coefficients. $k_{1}$ and $k_{2}$ are the modal stiffnesses of two flexural modes of HFVRG ($K_{F}$ herein). $k_{3}$ and $k_{4}$ are the modal stiffnesses of two translation modes of the design ($K_{T}$ herein). $K_{ri}(i=1,2)$ are the equivalent stiffnesses of ring structures associated with two flexural modes. $K_{hs}$, $K_{vs}$, and $K_{45}$ are the horizontal stiffness, the vertical stiffness and the stiffness along to the 45° direction of the semicircular elastic support beam, respectively. As shown in Figure 4. E is the elastic modulus of the silicon material. I and $I_{s}$ represent respectively the second moment of area of the ring cross-section and support beam cross-section. It is necessary to point out that the (111) silicon wafer was found to be in-plane isotropic in the literature [36,37]. $r_{r}$ and $r_{s}$ are the radius of the ring structure and semicircular elastic support beam, respectively. Mentioning that the pairs of modal masses and modal stiffnesses are identical if the ring gyroscope is uniform and axisymmetric (i.e., ideally fabricated). In addition, $K_{dsy1}$ and $K_{dsy1}$ are the stiffness of the drive/sense beam near and away from the resonant ring, respectively. $K_{hx}$ is the stiffness of the hinge beam.

### 3.3. The Stiffness Analysis of Drive/Sense Beam and Hinge Beam

The elastic beams with a large length and relatively small flexural strength such as hinge beams and drive/sense beams can be analyzed using a lumped-parameter model. On the other hand, the drive/sense beams and hinge beams in the design are U-shaped in series or in parallel, which can reduce their axial stress to some extent. Hence, there is a linear relationship between force and displacement. Energy principles in structural mechanics will be used to derive the stiffness of hinge beams and drive/sense beams.

**Figure 5:**
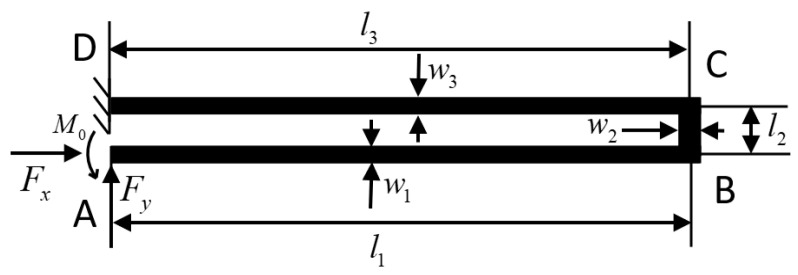
Structure schematic diagram of drive/sense beam.

The drive/sense beam is composed of three rectangular beams, as shown in Figure 5. The stiffness characteristics of drive/sense beams are explored using the Castigliano’s second theorem and the derivation is described in detail in Appendix A. The stiffness of drive/sense beam in the direction of x-axis and y-axis can be written as:

(12) $$K_{dsy}=\frac{E}{D},\;K_{dsx}=\frac{E}{H}$$

where

(13) $$D=\frac{\left(\begin{array}{l} I_{1}I_{2}l_{3}\left({\left(A+l_{2}\right)}^{2}C^{2}+\left(A+l_{2}\right)\left(2B-2l_{1}+l_{3}\right)C+l_{1}^{2}-\left(2B+l_{3}\right)l_{1}+l_{3}^{2}/3+l_{3}B+B^{2}\right)+\\ I_{1}l_{2}I_{3}\left(\left(A^{2}+l_{2}A+l_{2}^{2}/3\right)C^{2}+C\left(2A+l_{2}\right)\left(B-l_{1}\right)+{\left(B-l_{1}\right)}^{2}\right)+l_{1}I_{2}I_{3}\left(A^{2}C^{2}+AC\left(2B-l_{1}\right)+B^{2}-l_{1}B+l_{1}^{2}/3\right) \end{array}\right)}{I_{1}I_{2}I_{3}},\;\;H=\frac{\left(\begin{array}{l} I_{1}I_{2}l_{3}\left(\left(l_{1}^{2}-\left(2B+l_{3}\right)l_{1}+l_{3}^{2}/3+Bl_{3}+B^{2}\right)G^{2}+\left(A+l_{2}\right)\left(2B-2l_{1}+l_{3}\right)G+{\left(A+l_{2}\right)}^{2}\right)+\\ I_{1}l_{2}I_{3}\left({\left(B-l_{1}\right)}^{2}G^{2}+\left(2A+l_{2}\right)\left(B-l_{1}\right)G+\left(A^{2}+Al_{2}+l_{2}^{2}/3\right)\right)+l_{1}I_{2}I_{3}\left(\left(B^{2}-Bl_{1}+l_{1}^{2}/3\right)G^{2}+AG\left(2B-l_{1}\right)+A^{2}\right) \end{array}\right)}{I_{1}I_{2}I_{3}}\;C=-\frac{I_{1}I_{2}l_{3}\left(A+l_{2}\right)\left(B-l_{1}+l_{3}/2\right)+I_{1}l_{2}I_{3}\left(A+l_{2}/2\right)\left(B-l_{1}\right)+l_{1}I_{2}I_{3}A\left(B-l_{1}/2\right)}{I_{1}I_{2}l_{3}{\left(A+l_{2}\right)}^{2}+I_{1}l_{2}I_{3}\left(A^{2}+l_{2}A+l_{2}^{2}/3\right)+l_{1}I_{2}I_{3}A^{2}}\;G=-\frac{I_{1}I_{2}l_{3}\left(A+l_{2}\right)\left(B-l_{1}+l_{3}/2\right)+I_{1}l_{2}I_{3}\left(A+l_{2}/2\right)\left(B-l_{1}\right)+l_{1}I_{2}I_{3}A\left(B-l_{1}/2\right)}{I_{1}I_{2}l_{3}\left(l_{1}^{2}-\left(2B+l_{3}\right)l_{1}+B^{2}+Bl_{3}+l_{3}^{2}/3\right)+I_{1}l_{2}I_{3}{\left(B-l_{1}\right)}^{2}+l_{1}I_{2}I_{3}\left(B^{2}-Bl_{1}+l_{1}^{2}/3\right)}\;A=-\frac{I_{1}l_{2}\left(2I_{2}l_{3}+l_{2}I_{3}\right)}{2\left(I_{1}I_{2}l_{3}+I_{1}l_{2}I_{3}+l_{1}I_{2}I_{3}\right)},\;B=\frac{I_{1}I_{2}l_{3}\left(2l_{1}-l_{3}\right)+l_{1}I_{3}\left(2I_{1}l_{2}+l_{1}I_{2}\right)}{2\left(I_{1}I_{2}l_{3}+I_{1}l_{2}I_{3}+l_{1}I_{2}I_{3}\right)}$$

**Figure 6:**
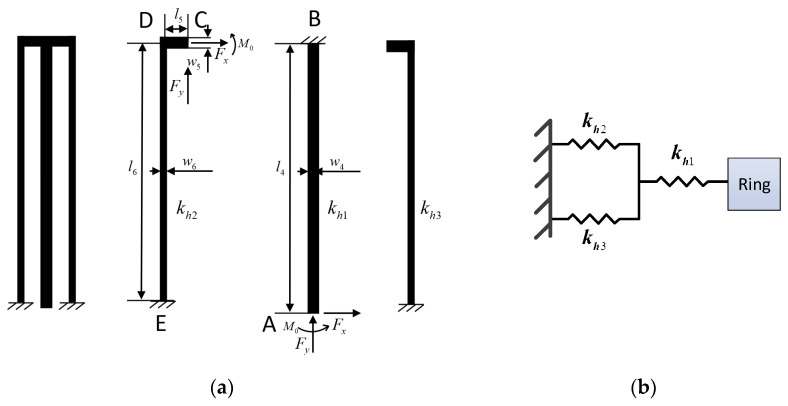
Structure schematic diagram (**a**) and lumped model (**b**) of the hinge beam.

Figure 6a displays the structure schematic diagram of the hinge beam. It can be seen that the hinge beam consists of three parts and can be simplified to the lumped model shown in Figure 6b, where the stiffness characteristics of $k_{h2}$ are exactly the same as those of $k_{h3}$. The stiffness characteristics of hinge beams are investigated using the same strategy as those of driving (or sense) beams. According to the previous analysis, the stiffness of the hinge beam in the direction of x-axis and y-axis can be written as:

(14) $$k_{hx}=\frac{2k_{h1x}k_{h2x}}{2k_{h2x}+k_{h1x}},\;k_{hy}=\frac{2k_{h1y}k_{h2y}}{2k_{h2y}+k_{h1y}}$$

where

(15) $$k_{h1x}=\frac{E}{D_{1}},\;k_{h1y}=\frac{E}{H_{1}},k_{h2x}=\frac{E}{D_{2}},\;k_{h2y}=\frac{E}{H_{2}}$$

(16) $$D_{1}=\frac{\left(A_{1}{}^{2}+A_{1}l_{4}+l_{4}^{2}/3\right)l_{4}}{I_{4}},\;H_{1}=0,\;A_{1}=-\frac{l_{4}}{2},\;A_{2}=\frac{I_{5}l_{6}^{2}}{2\left(I_{5}l_{6}+I_{6}l_{5}\right)},B_{2}=-\frac{2I_{5}l_{5}l_{6}+I_{6}l_{5}^{2}}{2\left(I_{5}l_{6}+I_{6}l_{5}\right)}\;D_{2}=\frac{\left(\begin{array}{l} \left(I_{5}l_{6}{\left(B_{2}+l_{5}\right)}^{2}+I_{6}l_{5}\left(B_{2}^{2}+l_{5}B_{2}+l_{5}^{2}/3\right)\right)C_{2}{}^{2}+\left(l_{6}A_{2}^{2}-l_{6}^{2}A_{6}+l_{6}^{3}/3\right)I_{5}\\ +I_{6}l_{5}A_{2}^{2}+\left(2I_{5}l_{6}\left(A_{2}-l_{6}/3\right)\left(B_{2}+l_{5}\right)+I_{6}l_{5}A_{2}\left(2B_{2}+l_{5}\right)\right)C_{2} \end{array}\right)}{I_{5}I_{6}}\;H_{2}=\frac{\left(\begin{array}{l} I_{5}l_{6}\left(l_{5}^{2}+l_{5}\left(2A_{2}G_{2}+2B_{2}-G_{2}l_{6}\right)+G_{2}^{2}l_{6}^{2}/3-l_{6}\left(B_{2}G_{2}+A_{2}G_{2}^{2}\right)+{\left(A_{2}G_{2}+B_{2}\right)}^{2}\right)\\ +I_{6}l_{5}\left({\left(A_{2}G_{2}+B_{2}\right)}^{2}+l_{5}\left(A_{2}G_{2}+B_{2}\right)+l_{5}^{2}/3\right) \end{array}\right)}{I_{5}I_{6}}\;C_{2}=-\frac{6I_{5}l_{6}A_{2}B_{2}+6I_{6}l_{5}A_{2}B_{2}+6I_{5}l_{5}l_{6}A_{2}+3I_{6}l_{5}^{2}A_{2}-3I_{5}l_{6}^{2}B_{2}-3I_{5}l_{5}l_{6}^{2}}{6B_{2}^{2}I_{5}l_{6}+6B_{2}^{2}I_{6}l_{5}+12B_{2}I_{5}l_{5}l_{6}+6B_{2}I_{6}l_{5}^{2}+6I_{5}l_{5}^{2}l_{6}+2I_{6}l_{5}^{3}}\;G_{2}=-\frac{6I_{5}l_{6}\left(B_{2}+l_{5}\right)\left(A_{2}-l_{6}/2\right)+3I_{6}l_{5}A_{2}\left(2B_{2}+l_{5}\right)}{\left(6l_{6}A_{2}^{2}-6l_{6}^{2}A_{2}+2l_{6}^{3}\right)I_{5}+6I_{6}l_{5}A_{2}^{2}}$$

### 3.4. Discussion of Resonant Frequency and FDR

The developed dynamical model concerning the HFVRG can be utilized to further predict the resonant frequencies of flexural modes and translation modes of the sensor. At typical working frequencies of the HFVRG, the angular acceleration term and centrifugal stiffness will not cause too much change and can be neglected. The natural frequencies for four normal modes are given by the simple relationships.

(17) $$f_{f}=\frac{1}{2\pi }\sqrt{\frac{k_{i}}{M_{i}}}\left(i=1,2\right),\;f_{t}=\frac{1}{2\pi }\sqrt{\frac{k_{i}}{M_{i}}}\left(i=3,4\right)$$

where $f_{f}$ and $f_{t}$ are the resonant frequencies of the flexural modes and translation modes in HFVRG, respectively.

In order to further investigate the optimization effect of each key parameter (mass parameter and stiffness parameter) on the mode ordering, the dimensionless parameter η is defined by:

(18) $$\eta =\frac{f_{t}-f_{f}}{f_{f}}$$

where η is defined as the frequency difference ratio (FDR) denoting the ratio of the frequency difference between translation and flexural modes to the resonant frequency of flexural mode.

Substituting Equations (8), (10) and (17) into Equation (18), we obtain:

(19) $$\eta =\sqrt{\frac{\left(k_{ns}+k_{ts}\right)\left(5m_{r}+32m_{f}\right)}{8\left(k_{r}+2k_{ns}\right)\left(m_{r}+2m_{f}\right)}}-1$$

where $k_{ns}=2K_{ns}+4K_{dsy1}+4K_{dsy2}$ and $k_{ts}=2K_{ts}+2K_{hx}+4K_{45}$ represent the normal stiffness and tangential stiffness of the whole structure, respectively. $k_{r}=K_{ri}$ and $m_{r}$ represent modal stiffness of resonant ring associated with two flexural modes and the mass of the ring structure, respectively.

It can be seen that the FDR is mainly determined by the stiffness parameters $k_{ns}$, $k_{ts}$ and $k_{r}$ as well as mass parameters $m_{r}$ and $m_{f}$. By solving the partial derivative of function η with respect to these variables, we obtain that:

(20) $$\frac{\partial \eta }{\partial k_{ns}}=\frac{\sqrt{2\left(5m_{r}+32m_{f}\right)}\left(k_{r}-2k_{ts}\right)}{8\sqrt{\left(k_{ns}+k_{ts}\right)\left(m_{r}+2m_{f}\right){\left(k_{r}+2k_{ns}\right)}^{3}}}$$

(21) $$\frac{\partial \eta }{\partial k_{ts}}=\frac{\sqrt{2\left(5m_{r}+32m_{f}\right)}}{8\sqrt{\left(k_{ns}+k_{ts}\right)\left(m_{r}+2m_{f}\right)\left(k_{r}+2k_{ns}\right)}}$$

(22) $$\frac{\partial \eta }{\partial k_{r}}=-\frac{\sqrt{2\left(k_{ns}+k_{ts}\right)\left(5m_{r}+32m_{f}\right)}}{\sqrt{\left(m_{r}+2m_{f}\right){\left(k_{r}+2k_{ns}\right)}^{3}}}$$

(23) $$\frac{\partial \eta }{\partial m_{r}}=-\frac{11m_{f}\sqrt{2\left(k_{ns}+k_{ts}\right)}}{4\sqrt{\left(5m_{r}+32m_{f}\right){\left(m_{r}+2m_{f}\right)}^{3}\left(k_{r}+2k_{ns}\right)}}$$

(24) $$\frac{\partial \eta }{\partial m_{f}}=\frac{11m_{r}\sqrt{2\left(k_{ns}+k_{ts}\right)}}{4\sqrt{\left(5m_{r}+32m_{f}\right){\left(m_{r}+2m_{f}\right)}^{3}\left(k_{r}+2k_{ns}\right)}}$$

In general, the values of the stiffness parameters $k_{ns}$, $k_{ts}$ and $k_{r}$ as well as mass parameters $m_{r}$ and $m_{f}$ are greater than 0. From Equations (21)–(24), we can find that:

(25) $$\frac{\partial \eta }{\partial k_{ts}}>0,\;\frac{\partial \eta }{\partial k_{r}}<0,\;\frac{\partial \eta }{\partial m_{r}}<0,\;\frac{\partial \eta }{\partial m_{f}}>0$$

From Equation (25), the FDR η monotonically increases with increasing $k_{ts}$ and $m_{f}$, but decreases with increasing $k_{r}$ and $m_{r}$. Within a reasonable range, $k_{r}$ and $m_{r}$ are as small as possible and $k_{ts}$ and $m_{f}$ are as large as possible; the FDR can be improved effectively and the effect of translation vibration on the performance of ring gyroscope can be eliminated effectively.

From Equation (20), it appears that the influence of the normal stiffness of the whole device on FDR depends on the difference between the flexural modal stiffness of resonant ring and the two times tangential stiffness of the design. Therefore, we conclude that under the premise of $k_{r}-2k_{ts}<0$, the FDR decreases with increasing the normal stiffness of the whole device.

The aforementioned VRG with different forms of supporting beams often provided poor modal ordering. It can be remarked that the hinge-frame mechanism we proposed can change the modal ordering of original ring gyroscope and is expected to be applied to all kinds of existing ring gyroscopes. Here, four architectures based on HFVRG are designed to explore the impact of the parameters of hinge-frame mechanism such as $K_{hx}$, $K_{dsy}$ and $m_{f}$ on FDR, defined as types A, B, C, and D. The $K_{hx}$ of type B, the $m_{f}$ of type C and the $K_{dsy}$ of type D are slightly larger than those of type A. This can be achieved by intentionally decreasing the spring width of the hinge beam and drive/sense beam as well as the volume of drive/sense frame, respectively.

### 3.5. Theoretical Calculation

The critical parameters of the geometric and material properties of the HFVRG in the designs and FEM simulation stage are shown in Table 1 and Table 2, respectively.

These parameters are substituted into the previous equation, and the main theoretical stiffness, theoretical resonant frequencies of flexural modes and translation modes as well as FDR in all types can be obtained, as indicated in Table 3.

It can be seen that the theoretical resonant frequency of flexural mode is lower than that of translation mode in all types, which results in an excellent mode ordering. Furthermore, it is necessary to point out that the FDR of type A is higher than that of type B and C, and lower than that of type D from Table 3, which is consistent with previous analysis.

## 4. FEM Simulation and Analysis

### 4.1. Analysis of Various Beams

**Figure 7:**
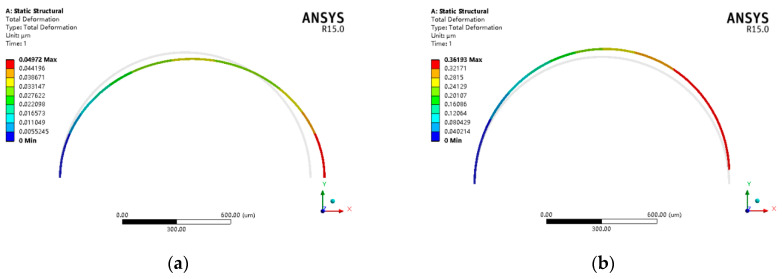

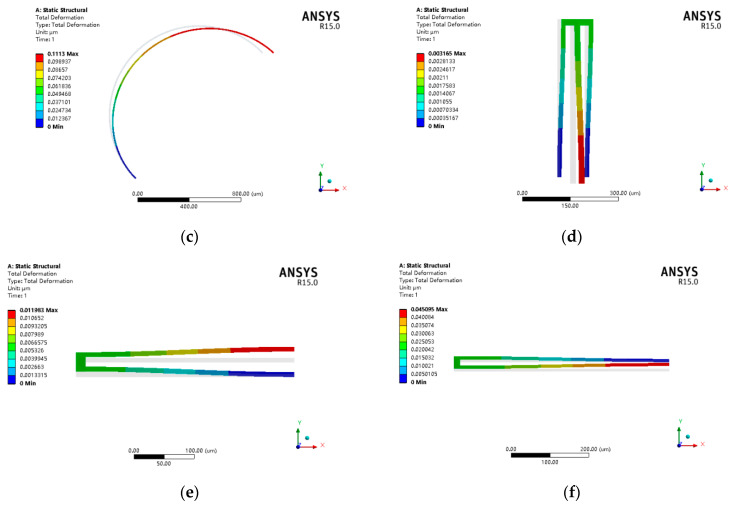
Stiffness of various beams of MEMS gyroscope: (**a**) deformation of $K_{ns}$; (**b**) deformation of $K_{ts}$; (**c**) deformation of $K_{45}$; (**d**) deformation of $K_{hx}$; (**e**) deformation of $K_{dsy1}$; (**f**) deformation of $K_{dsy2}$.

From Equation (20), the modal stiffness is dependent on the stiffness of various beams in the designs. Therefore, simulations are carried out on the stiffness of these beams by applying a 1 μN force in the linked structure, as shown in Figure 7. The normal stiffness, tangential stiffness and the stiffness along to the 45° support beam are shown in Figure 7a–c, respectively. The stiffness of the hinge beam and the two drive/sense beams are shown in Figure 7d–f, respectively. Using the formula k=F/x, the stiffness can be obtained:

(26) $$K_{ns}=\frac{1\;\mu N}{0.04972\;\mu m}=20.11\;N/m,\;K_{ts}=\frac{1\;\mu N}{0.36193\;\mu m}=2.76\;N/m\;K_{45}=\frac{1\;\mu N}{0.1113\;\mu m}=8.98\;N/m,\;K_{hx\_A\_C\_D}=\frac{1\;\mu N}{0.003165\;\mu m}=315.96\;N/m\;K_{hx\_B}=\frac{1\;\mu N}{0.004009\;\mu m}=249.44\;N/m,\;K_{dsy1\_A\_B\_C}=\frac{1\;\mu N}{0.011983\;\mu m}=83.45\;N/m\;K_{dsy1\_D}=\frac{1\;\mu N}{0.017716\;\mu m}=56.45\;N/m,\;K_{dsy2}=\frac{1\;\mu N}{0.045095\mu m}=22.18\;N/m$$

It can be seen that the stiffness of various beams by FEM from Equation (26) and Figure 7 is basically in accordance with the theoretical results depicted in Table 3, which verifies the proposed theoretical model.

### 4.2. Modal Analysis and Comparisons

Modal analysis can be applied to investigate the vibration characteristics of the device structure, such as the mode shape, resonant frequency and vibration stability. A more detailed structural model simulation is carried out by FEM using the commercial software ANSYS-Workbench. The mesh element is SOLID186, which is a high-order three-dimensional 20-node solid structure unit. All the structures use hexahedral meshes: the linear beam uses a small mesh, the drive/sense frames use slightly larger meshes, and all anchor uses larger hexahedral meshes. Types A, B, C, and D have a total of 586,872, 597,224, 586,040, and 587,704 nodes, respectively. The main parameters of the structures and the material properties of silicon are shown in Table 1 and Table 2, respectively.

**Figure 8:**
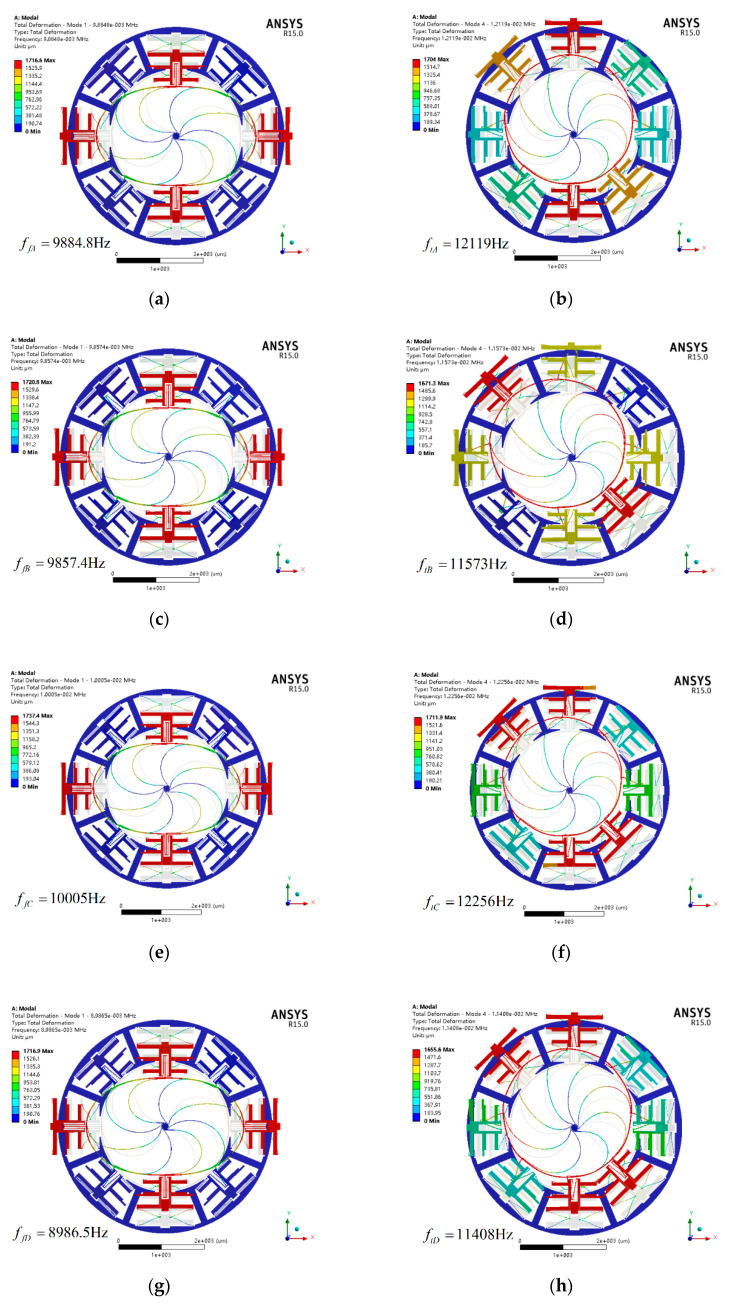
Flexural modal diagram (**a**), (**c**), (**e**), (**g**) and translation modal diagram (**b**), (**d**), (**f**), (**h**) of type A, B, C, and D.

Figure 8 shows the flexural modal diagram and translation modal diagram of all types. It is necessary to point out that the two flexural modes of all types are the first two order modes and the two translation modes of them are the third and fourth modes. It can be seen that minor adjustment of structural parameters for hinge-frame mechanism cannot cause a change in the mode ordering. Besides, this design can effectively improve the resonant frequency of disturbance modes such as torsional mode, out-of-plane translation mode and out-of-plane rocking mode compared with the original one. The natural frequencies of the flexural and translation modes and the FDR of all types are listed in Table 4.

It can be seen from Table 4 that the FDR of type A is higher than that of type B and C, and is lower than that of type D, which is in keeping with theoretical results in Section 3.5. The theoretical and simulation values of all types and the corresponding error rates are obtained and listed in Table 5.

From Table 5, it can be found that the simulation results are consistent with the theoretical values in all types. In addition, the frequency of flexural mode is lower than that of translation mode in each type. Therefore, the proposed theoretical model is accurate and rational for the hinge-frame mechanism.

### 4.3. Nonlinear and Coupling Analysis

One of the driving and detecting errors neglected in the above simplified model is derived from the structure nonlinearity, which is caused by the large deformation of drive or sense beams. Therefore, a validation is provided here. Since most of the literatures have pointed out that the dominant signal in output signals is the quadrature error, it is necessary to further validate the advantage of decoupling and high linearity in the design with a simple analysis by FEM using ANSYS-Workbench.

**Figure 9:**
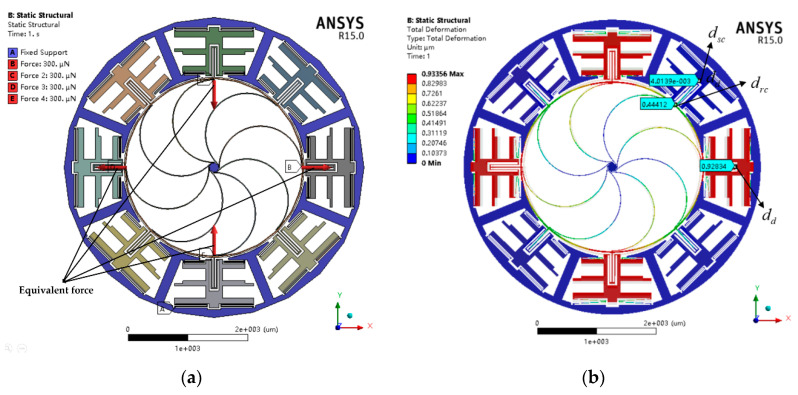
The predefined environments (**a**) and total deformation with a magnification of 50 times (**b**) when the equivalent force is 300  μN of nonlinear analysis.

The nonlinear analysis can be used to analyze problems where the stress–strain relationship of the material is nonlinear and check whether the model gives reasonable results or not. In this simulation, the parameters of the structure, material properties of silicon, mesh division and element type are the same as those of the modal analysis. The predefined environments (i.e., supports and loads) are shown in Figure 9a. It can be seen that all the anchors are set to the fixed support, and the equivalent force of 300  μN is applied at the antinode on the ring to simulate the deformation of the flexural mode. The total deformation with a magnification of 50 times is depicted in Figure 9b. We define that $d_{d}$ is the displacement of drive frame, $d_{sc}$ is the coupling displacement of sense frame and $d_{rc}$ is the coupling displacement of node on resonant ring. For comparison purposes, $d_{rc}$ is represented as the coupling displacement of sense electrodes in typical VRG.

**Figure 10:**
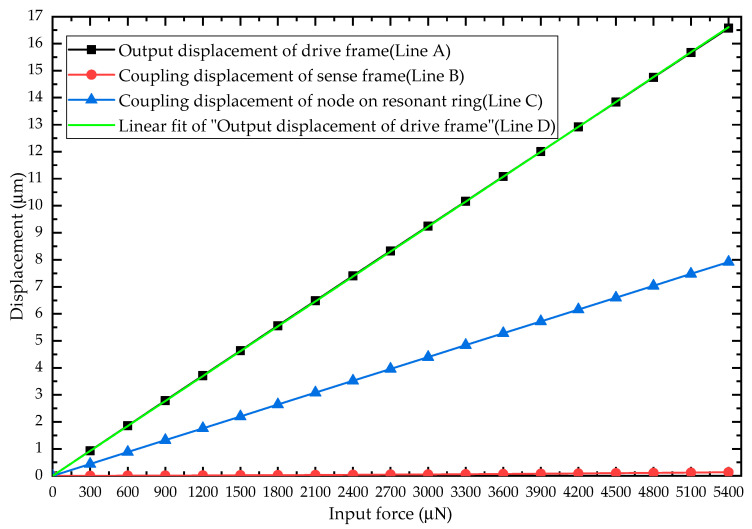
The output displacement of drive frame (Line A), sense frame (Line B) and node of resonant ring (Line C) at different input forces as well as linear fit of measured data (Line D).

From Table 1. It can be seen that the interval of drive/sense U-shape beam has a width of 16  μm ($l_{21}$). Therefore, the maximum displacement of the whole structure is 16  μm. After nonlinear analysis, the input equivalent force vs. output displacement of drive frame ($d_{d}$), sense frame ($d_{sc}$) and node of resonant ring ($d_{rc}$) as well as linear fit of measured data are shown in Figure 10.

As can be seen from Figure 10, the displacement of drive frame (Line A) and the node on resonant ring increases (Line C) almost linearly with the increase of the input force, and the displacement of drive frame (Line A) is about twice that of the node on the resonant ring (Line C). However, the displacement of sense frame (Line B) remains at a low level though it has a tendency to increase, which verifies that the design has an advantage of quadrature decoupling compared with the typical VRG. Additionally, in order to verify the linearity of the HFVRG in the maximum working range, the deviation from the best-fit line (Line D) is calculated. The structure nonlinearity caused by large deformation of drive or sense beams is found to be negligible (adjusted R^2^ = 0.99999), which verifies that the design works within a linear interval.

## 5. Discussion

It is noteworthy that there are still some errors in the theoretical results and simulation results of the resonant frequencies for both translation and flexural modes. This is due to the assumption that drive/sense beam as a one-dimensional linear spring in theoretical analysis. However, these errors have no effect on the final modal characteristics.

The present work is fundamental for the novel HFVRG, which focuses on the structure design and principle verification. Further research includes: (1) The investigation of high shock reliability will pay more attention to VRG architecture. (2) The proposed HFVRG will be fabricated through a conventional silicon-on-glass (SOG) and deep reactive ion etching (DRIE) process for experimental verification.

## 6. Conclusions

In this study, a high-linearity, low quadrature error and prefect mode ordering MEMS vibrating ring gyroscope with a hinge-frame mechanism is proposed. A systematic dynamic model is established to investigate the modal characteristics, and the FDR is introduced to characterize the optimization effect. The theoretical calculation results show that the FDR increases with increased tangential stiffness of the whole structure, while the mass of drive/sense frame decreases with increased normal stiffness of the whole structure under certain conditions. Four architectures based on HFVRG with different values of stiffness or mass are designed. In addition, the stiffness of all the four types of beams and modal analysis are carried out by FEM simulation, which demonstrates that the stiffness and modal characteristics of the design are in keeping with theoretical results. Furthermore, the nonlinearity analysis indicates that the HFVRG possesses high-linearity and decoupling characteristics. Consequently, the proposed HFVRG is capable to offer a high linearity excitation and detection strategy, improved mode ordering and eliminate the quadrature coupling effectively.

## Figures and Tables

**Table 1:** Main parameters of the structures.

Parameter	Value
Radius of resonant ring in all types ($r_{r}$) (μm)	1500
Width of resonant ring in all types ($w_{r}$) (μm)	25
Radius of support beam in all types ($r_{s}$) (μm)	690
Width of support beam in all types ($w_{r}$) (μm)	12
Length of drive/sense beam 1 in type A, B and C [$l_{11},l_{21},l_{31}$] (μm)	[345,16,345]
Width of drive/sense beam 1 in type A, B and C [$w_{11},w_{21},w_{31}$] (μm)	[8,16,8]
Length of drive/sense beam 1 in type D [$w_{11},w_{21},w_{31}$] (μm)	[345,18,345]
Width of drive/sense beam 1 in type D [$w_{11},w_{21},w_{31}$] (μm)	[7,16,7]
Length of drive/sense beam 2 in all types [$l_{12},l_{22},l_{32}$] (μm)	[537,16,537]
Width of drive/sense beam 2 in all types [$w_{12},w_{22},w_{32}$] (μm)	[8,16,8]
Length of hinge beam in type A, C and D [$l_{4},l_{5},l_{6}$] (μm)	[505,30,485]
Width of hinge beam in type A, C and D [$w_{4},w_{5},w_{6}$] (μm)	[18,20,14]
Length of hinge beam in type B [$l_{4},l_{5},l_{6}$] (μm)	[505,32,485]
Width of hinge beam in type B [ $w_{4},w_{5},w_{6}$] ( μm)	[18,20,12]
Height of the whole structure in all types (h) (μm)	80
Mass of resonant ring in all types ($m_{r}$) (kg)	$4.35\times 10^{-8}$
Mass of drive/sense frame in type A, B and D ($m_{f}$) (kg)	$6.94\times 10^{-8}$
Mass of drive/sense frame in type C ($m_{f}$) (kg)	$6.73\times 10^{-8}$

**Table 2:** Material parameters for FEM simulation.

Parameter	Young’s Modulus (Pa)	Poisson’s Ratio	Density ( ${\mathrm{kg}/m}^{3}$)
Values	$1.7\times 10^{11}$	0.28	2330

**Table 3:** Main theoretical stiffness and theoretical resonant frequencies.

Parameter	Type A	Type B	Type C	Type D
Stiffness of resonant ring ($K_{r}$) (N/m)	164.75			
Normal stiffness of support beam ($K_{ns}$) (N/m)	20.13			
Tangential stiffness of support beam ($K_{ts}$) (N/m)	3.80			
The stiffness along to the 45° of support beam ($K_{45}$) (N/m)	11.96			
Stiffness of drive/sense beam 1 ($K_{dsy1}$) (N/m)	84.06	84.06	84.06	56.25
Stiffness of drive/sense beam 2 ($K_{dsy2}$) (N/m)	22.36			
Stiffness of hinge beam ($K_{hx}$) (N/m)	314.82	245.29	314.82	314.82
Resonant frequency of flexural mode of HFVRG ($f_{f}$) (Hz)	9551	9551	9685	8528
Resonant frequency of translation mode of HFVRG ($f_{t}$) (Hz)	12,652	11,863	12,800	12,025
Frequency difference ratio (FDR) (η)	32.46%	24.20%	32.16%	41.01%

**Table 4:** Natural frequencies of the flexural and translation modes and the FDR of all types.

Parameter	Type A	Type B	Type C	Type D
Resonant frequency of flexural mode (Hz)	9884.8	9857.4	10,005	8986.5
Resonant frequency of translation mode (Hz)	12,119	11,573	12,256	11,408
Frequency difference ratio (FDR)	22.60%	17.40%	22.50%	26.95%

**Table 5:** Comparison of theoretical and simulation values of types A, B, C, and D.

Type	Mode Shape	Theoretical Value	Simulation Value	Error Rate
Type A	Flexural mode	9551	9884.8	−3.50%
	Translation mode	12,652	12,119	4.21%
Type B	Flexural mode	9551	9857.4	−3.21%
	Translation mode	11,863	11,573	2.44%
Type C	Flexural mode	9685	10,005	−3.40%
	Translation mode	12,800	12,256	4.25%
Type D	Flexural mode	8528	8986.5	−5.38%
	Translation mode	12,025	11,408	5.13%

## Appendix A

For this analysis the drive/sense beams are divided into three sections: AB, BC and CD. The applied torque $M_{0}$ provided by the drive/sense frame on the left side of AB segment will constrain the rotation of the beam from Figure 5. The torques of each section of the elastic beam can be obtained by establishing a local rectangular coordinate system (ξ−δ) for the three sections.

(A1) $$\mathrm{AB}\;\sec\mathrm{tion}:M_{1}=M_{0}-F_{y}\cdot \xi$$

(A2) $$\mathrm{BC}\;\sec\mathrm{tion}:M_{2}=M_{0}-F_{y}\cdot l_{1}+F_{x}\cdot \xi$$

(A3) $$\mathrm{CD}\;\sec\mathrm{tion}:M_{3}=M_{0}-F_{y}\cdot \left(l_{3}-\xi \right)+F_{x}\cdot l_{2}$$

where ξ is the independent variable of the local coordinate system.

According to elastic mechanics and material mechanics, the strain energy of the entire drive/sense beam can be obtained.

(A4) $$U=\int_{0}^{l_{1}}\frac{M_{1}^{2}}{2EI_{1}}d\xi +\int_{0}^{l_{2}}\frac{M_{2}^{2}}{2EI_{2}}d\xi +\int_{0}^{l_{3}}\frac{M_{3}^{2}}{2EI_{3}}d\xi$$

The elastic stiffness of drive/sense beam in the direction of y-axis will be explored through Castigliano’s second theorem, we assume that a force is applied in the y-axis. At this point, the angle and the displacement along the x-axis of the end of the elastic beam (point A) are both 0. We can obtain that:

(A5) $$\theta_{z}=\frac{\partial U}{\partial M_{0}}=\int_{0}^{l_{1}}\frac{M_{1}}{EI_{1}}\frac{\partial M_{1}}{\partial M_{0}}d\xi +\int_{0}^{l_{2}}\frac{M_{2}}{EI_{2}}\frac{\partial M_{2}}{\partial M_{0}}d\xi +\int_{0}^{l_{3}}\frac{M_{3}}{EI_{3}}\frac{\partial M_{3}}{\partial M_{0}}d\xi =0$$

(A6) $$\delta_{x}=\frac{\partial U}{\partial F_{x}}=\int_{0}^{l_{1}}\frac{M_{1}}{EI_{1}}\frac{\partial M_{1}}{\partial F_{x}}d\xi +\int_{0}^{l_{2}}\frac{M_{2}}{EI_{2}}\frac{\partial M_{2}}{\partial F_{x}}d\xi +\int_{0}^{l_{3}}\frac{M_{3}}{EI_{3}}\frac{\partial M_{3}}{\partial F_{x}}d\xi =0$$

Substituting Equation (A1), (A2) and (A3) into Equations (A5), we obtain:

(A7) $$M_{0}=AF_{x}+BF_{y}$$

where

(A8) $$A=-\frac{I_{1}l_{2}\left(2I_{2}l_{3}+l_{2}I_{3}\right)}{2\left(I_{1}I_{2}l_{3}+I_{1}l_{2}I_{3}+l_{1}I_{2}I_{3}\right)},\;B=\frac{I_{1}I_{2}l_{3}\left(2l_{1}-l_{3}\right)+l_{1}I_{3}\left(2I_{1}l_{2}+l_{1}I_{2}\right)}{2\left(I_{1}I_{2}l_{3}+I_{1}l_{2}I_{3}+l_{1}I_{2}I_{3}\right)}$$

Substituting Equation (A7) into Equations (A1), (A2) and (A3), we obtain:

(A9) $$\left\{ \begin{array}{l} M_{1}=AF_{x}+\left(B-\xi \right)F_{y}\\ M_{2}=\left(A+\xi \right)F_{x}+\left(B-l_{1}\right)F_{y}\\ M_{3}=\left(A+l_{2}\right)F_{x}+\left(B-l_{3}+\xi \right)\cdot F_{y} \end{array}\right.$$

Substituting Equation (A9) into Equations (A6), we obtain:

(A10) $$F_{x}=CF_{y}$$

where

(A11) $$C=-\frac{I_{1}I_{2}l_{3}\left(A+l_{2}\right)\left(B-l_{1}+l_{3}/2\right)+I_{1}l_{2}I_{3}\left(A+l_{2}/2\right)\left(B-l_{1}\right)+l_{1}I_{2}I_{3}A\left(B-l_{1}/2\right)}{I_{1}I_{2}l_{3}{\left(A+l_{2}\right)}^{2}+I_{1}l_{2}I_{3}\left(A^{2}+l_{2}A+l_{2}^{2}/3\right)+l_{1}I_{2}I_{3}A^{2}}$$

Substituting Equation (A10) into Equations (A9), we obtain:

(A12) $$\left\{ \begin{array}{l} M_{1}=\left(AC+B-\xi \right)F_{y}\\ M_{2}=\left(AC+C\xi +B-l_{1}\right)F_{y}\\ M_{3}=\left(AC+Cl_{2}+B-l_{3}+\xi \right)F_{y} \end{array}\right.$$

The Castigliano’s second theorem is used to calculate the displacement in the direction of *y*-axis at point A.

(A13) $$\delta_{y}=\frac{\partial U}{\partial F_{y}}=\int_{0}^{l_{1}}\frac{M_{1}}{EI_{1}}\frac{\partial M_{1}}{\partial F_{y}}d\xi +\int_{0}^{l_{2}}\frac{M_{2}}{EI_{2}}\frac{\partial M_{2}}{\partial F_{y}}d\xi +\int_{0}^{l_{3}}\frac{M_{3}}{EI_{3}}\frac{\partial M_{3}}{\partial F_{y}}d\xi$$

Substitute Equation (A12) into Equation (A13) and simplify it, we can obtain that:

(A14) $$\delta_{y}=\frac{F_{y}D}{E}$$

where

(A15) $$D=\frac{\left(\begin{array}{l} I_{1}I_{2}l_{3}\left({\left(A+l_{2}\right)}^{2}C^{2}+\left(A+l_{2}\right)\left(2B-2l_{1}+l_{3}\right)C+l_{1}^{2}-\left(2B+l_{3}\right)l_{1}+l_{3}^{2}/3+l_{3}B+B^{2}\right)+\\ I_{1}l_{2}I_{3}\left(\left(A^{2}+l_{2}A+l_{2}^{2}/3\right)C^{2}+C\left(2A+l_{2}\right)\left(B-l_{1}\right)+{\left(B-l_{1}\right)}^{2}\right)+l_{1}I_{2}I_{3}\left(A^{2}C^{2}+AC\left(2B-l_{1}\right)+B^{2}-l_{1}B+l_{1}^{2}/3\right) \end{array}\right)}{I_{1}I_{2}I_{3}}$$

The elastic stiffness of drive/sense beam in the direction of *y*-axis can be obtained.

(A16) $$K_{dsy}=\frac{E}{D}$$

The same method is used to derive the elastic stiffness of drive/sense beam in the direction of *x*-axis.

(A17) $$K_{dsx}=\frac{E}{H}$$

where

(A18) $$H=\frac{\left(\begin{array}{l} I_{1}I_{2}l_{3}\left(\left(l_{1}^{2}-\left(2B+l_{3}\right)l_{1}+l_{3}^{2}/3+Bl_{3}+B^{2}\right)G^{2}+\left(A+l_{2}\right)\left(2B-2l_{1}+l_{3}\right)G+{\left(A+l_{2}\right)}^{2}\right)+\\ I_{1}l_{2}I_{3}\left({\left(B-l_{1}\right)}^{2}G^{2}+\left(2A+l_{2}\right)\left(B-l_{1}\right)G+\left(A^{2}+Al_{2}+l_{2}^{2}/3\right)\right)+l_{1}I_{2}I_{3}\left(\left(B^{2}-Bl_{1}+l_{1}^{2}/3\right)G^{2}+AG\left(2B-l_{1}\right)+A^{2}\right) \end{array}\right)}{I_{1}I_{2}I_{3}}$$

(A19) $$G=-\frac{I_{1}I_{2}l_{3}\left(A+l_{2}\right)\left(B-l_{1}+l_{3}/2\right)+I_{1}l_{2}I_{3}\left(A+l_{2}/2\right)\left(B-l_{1}\right)+l_{1}I_{2}I_{3}A\left(B-l_{1}/2\right)}{I_{1}I_{2}l_{3}\left(l_{1}^{2}-\left(2B+l_{3}\right)l_{1}+B^{2}+Bl_{3}+l_{3}^{2}/3\right)+I_{1}l_{2}I_{3}{\left(B-l_{1}\right)}^{2}+l_{1}I_{2}I_{3}\left(B^{2}-Bl_{1}+l_{1}^{2}/3\right)}$$
